# Influence of UV nail lamps radiation on human keratinocytes viability

**DOI:** 10.1038/s41598-023-49814-7

**Published:** 2023-12-18

**Authors:** Anna Słabicka-Jakubczyk, Miłosz Lewandowski, Paulina Pastuszak, Wioletta Barańska-Rybak, Magdalena Górska-Ponikowska

**Affiliations:** 1 Department of Medical Chemistry, Medical University of Gdansk, Debinki 1, 80-211 Gdansk, Poland; 2 Department of Dermatology, Venereology and Allergology, Faculty of Medicine, Medical University of Gdansk, Smoluchowskiego 17, 80-214 Gdansk, Poland; 3 Department of Biophysics, Institute of Biomaterials and Biomolecular Systems, University of Stuttgart, Stuttgart, Germany; 4 Euro-Mediterranean Institute of Science and Technology, Palermo, Italy

**Keywords:** Cell biology, Chemical biology, Health care, Medical research, Molecular medicine

## Abstract

Ultraviolet nail lamps are becoming increasingly popular, however, the safety of their use remains controversial. The following article directly responds to recently published literature data and aims to determine the viability of human keratinocytes irradiated by a UV nail-drying machine. Cells were exposed to 365–405 nm wavelength UV light emitted by a nail drying machine in two time variants: 4 and 20 min, with and without sunscreen cream SPF50 protection, and compared to the untreated control. Compared to the control, cell viability after irradiation for 4 min decreased insignificantly (*p* < 0.1), however for 20 min decreased by 35% (*p* < 0.0001). Furthermore, cells with sunscreen protection compared to those without showed significantly increased viability, regardless of time-variant (*p* < 0.0001). The study shows that 4-min irradiation does not significantly reduce the viability of human keratinocytes and the time of 20 min significantly alters the research results compared to 4 min, which corresponds to real conditions. The results suggest that typical manicure exposure time does not significantly affect keratinocyte viability, which could increase the risk of developing skin cancers. Despite the above results, it is recommended to use sunscreen protection on your hands during the procedure, which significantly increases the viability of keratinocytes during ultraviolet nail lamp radiation.

## Introduction

For both personal and professional nail care, ultraviolet (UV) nail lamps, a source of artificial UV light , are becoming increasingly popular^1,2^. In 2010–2011, over 87% of nail salons reported using a UV light (2010–2011, Industry Statistics)^1^. Some manicure techniques such as acrylic or dip powder are based on chemical curing, while others such as acrylic nails, UV top sealers, or top coats are light-curing materials that require UV to initiate the curing process^2,3^. Consequently, the current state of affairs raises concerns about the mutagenic potential of various types of UV lamps and their contribution to the development of skin cancer. Scientists' interest in the following topic is even more justified considering the impact of UV light on skin cells. Although the mechanisms leading to the development of non-melanoma skin cancers (NMSC), derived from epidermal keratinocytes, and melanoma are multifactorial, UV is a very important factor in their development, leading to DNA damage and the development of somatic mutations, inflammation, oxidative stress and defective activity of immune cells^4,5^.

Products cured in UV lamps became popular in the early 1990s^3,6^. They change their state of aggregation thanks to the presence of photoinitiators that absorb the UVA lightproduced by manicure lamps and dissolve, releasing free radicals that initiate the polymerization process^7^. In order for the reaction to be complete and correct, the duration of irradiation and the lamp power should be adjusted to the manufacturer's recommendations and the formulation of a given product.

Two types of lamps are currently used in beauty salons: light emitting diode (LED) lamps and UV nail lamps consisting of fluorescent bulbs. UV lamps, curing the product in 2–4 min emit UV from 300 to 410 nm, with a peak emission at 375 nm, while LED lamps, curing the product in 30–60 s, emit light with a peak wavelength of 385 nm and a wavelength range of 375 to 425 nm^1,8^. Due to the very large variety of products and techniques used, it is difficult to indicate one standard scheme of UV and LED lamp use. However, many sources indicate that during the procedure, the hands are radiated for around 3–6 min, and the sessions are usually repeated by clients every two or three weeks^9–12^.

In addition, in the context of the development of subungual melanoma, it should be noted that the nail plate completely blocks UVB light, and only minimally allows penetration of UVA light^13^.

There are still controversial reports about the safety of their use. The following article is a direct response to the article "DNA damage and somatic mutations in mammalian cells after irradiation with a nail polish dryer" published in Nature Communications on January 17, 2023, strongly suggesting that radiation emitted by UV-nail polish dryers may cause cancers of the hand and that UV-nail polish dryers, may increase the risk of early-onset skin cancer, being the opposite to many previously published studies, indicated low level of risk.

The aim of the study was to determine the viability of human keratinocytes, from which most common skin cancers—NMSC originate, irradiated by a UV nail-drying machine for 4 and 20 min, with and without sunscreen cream protection, compared to the control.

## Materials and methods

### Study design

**Figure 1:**
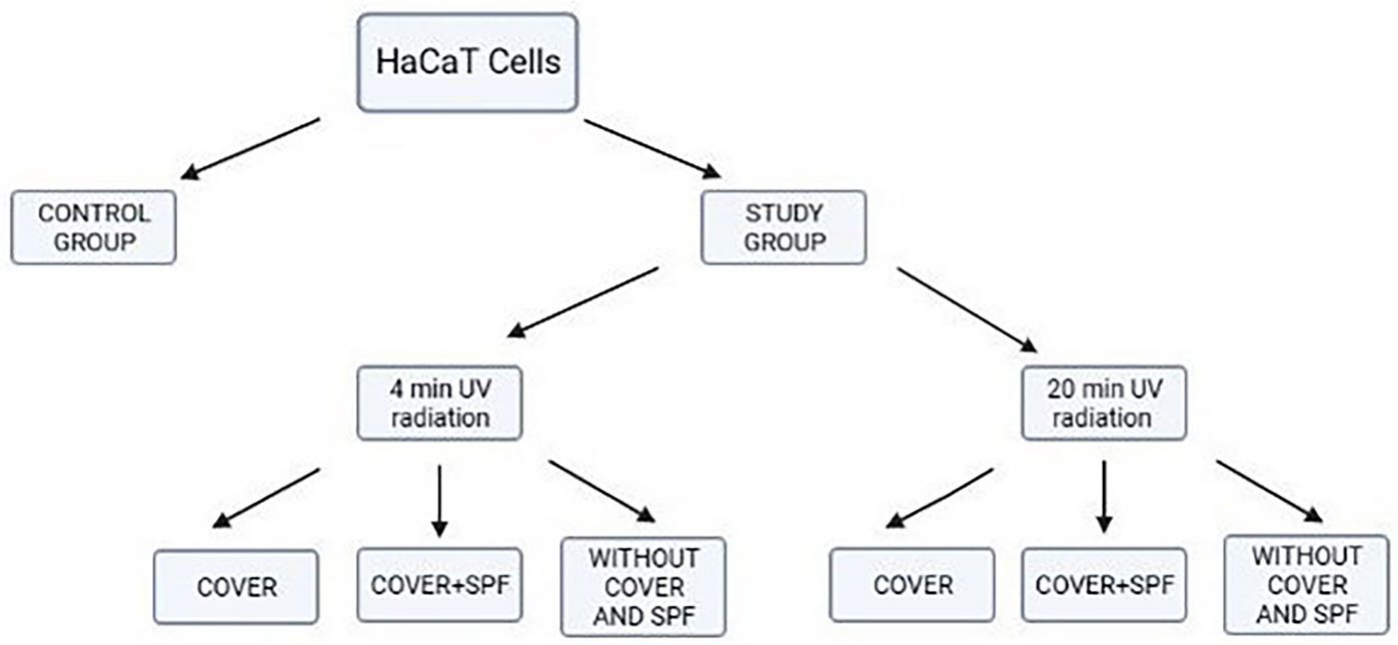
Study design.

The HaCaT human keratinocyte line was used to perform the study. Cells were grown in 96-well plates and exposed to UV light emitted by a nail drying machine in two-time variants: 4 and 20 min. Each time variant cells were divided into three 96-well plates: a plate covered with a polystyrene lid, a plate covered with a polystyrene lid and sunscreen cream, and a 96-well plate without a lid and sunscreen cream. (Fig. 1). As control group cells were not treated with UV lamp radiation. In order to evaluate the influence of UV light exposure on HaCaT cell viability, a 3-[4,5-dimethylthiazol 2-yl]-2,5-diphenyltetrazolium bromide (MTT) assay was performed. Polystyren lid used in the study was made from general polystyrene (GPPS) and its’ UV absorption according to study by Tong Li* in 365–405 nm range light is very low^14^. All methods were carried out in accordance with relevant guidelines.

### Cell line and cell culture

The human keratinocyte HaCaT cell line was obtained from the American Type Culture Collection (ATCC, Manassas, VA, USA). Dulbecco’s modified eagle medium (Sigma-Aldrich- D6046) supplemented with 10% fetal bovine serum (FBS), and 1% penicillin/streptomycin was used. The cells were cultured at 37 °C in a 5% CO_2_ atmosphere.

### UV-nail polish machine

24/48 W UV nail drying machine (model: SUNONE, SUN 1, UV/LED lamp, 24/48w power, Leobert, Poland) containing 48 LEDs were used. According to the device specification, the manufacturer declares it emits 365–405 nm range light. Calculated intensity of UV radiation is 10mW/cm2.

### Cell line treatment

To investigate the influence of UV-nail polish drying machine on HaCaT cell line viability. The cells were treated with a UV lamp for two times variants: 4 min and 20 min. During irradiation, control and treated cells were kept in a DMEM medium with the addition of FBS and penicillin/streptomycin. The distance that the UV lamp was from the cell plates during exposure was 4 cm. The first plate was UV irradiated and covered with a polystyrene lid. The irradiation with the cover was used to check whether it had any protective properties on the cells. Results revealed the lid has no significant protective action, therefore the second irritated plate was studied with a lid covered with 0,5 g of sunscreen cream (Holika Holika, Aloe Aoothing Essence, 50 SPF/PA +  +  +  + , Korea). The third plate was irradiated with a UV lamp without any lid and sunscreen cream. Cells irradiated for 20 min were examined according to the same methodology regimen as cells irradiated for 4 min. Cells were maintained at a temperature of approximately 20–25 degrees Celsius during exposure to UV. Control cells were not radiated and no sunscreen was applied to them. During the irradiation of the remaining samples, the control cells were kept outside the incubator in the dark for the same time as experimental conditions, 4 min and 20 min.

### Cell viability test (MTT test)

HaCaT cells were seeded on a 96-well plate at a density of 12,000 per well. After 24 h, the cells were exposed to the UV drying device. After treating the cells with UV light, 3-[4,5-dimethylthiazol 2-yl]-2,5-diphenyltetrazolium bromide (MTT) (M2128, Sigma-Aldrich, Poznań, Poland) was added at a concentration of 0.5 mg/mL. Subsequently, the plates were incubated for 2 h at 37 °C, and then 100 µL DMSO was added to dissolve the formazan crystals. Absorbances at 450 nm were read using a Jupiter reader (Asys Hitech, biogenet). Each experiment was repeated at least three times. MTT assay was used because of its’ high credibility, simplicity, and relatively quick to perform. In addition, it allows to demonstrate the viability of the cells immediately after irradiation.”

### Statistical analysis

Values are mean ± SE of three independent experiments. Data were analyzed with GraphPad Prism Software version 8.0.1 using bidirectional ANOVA with Tukey's multiple comparisons tests. A p-value of less than 0.05 was considered to be statistically significant. The data were evaluated using GraphPad Prism (GraphPad Software, Inc., Version 8 (USA, San Diego).

### Ethical approval

According to article 39 of the Act of 5 December 1996 on the medical profession in Poland the study does not require Bioethics Committee approval because the experiment was carried out without human participation and the cells used for it were not collected by us, but obtained from commercially available cell lines.

## Results

### In the case of treating HaCat cells for 4 min with UV light, the results were as followed

**Figure 2:**
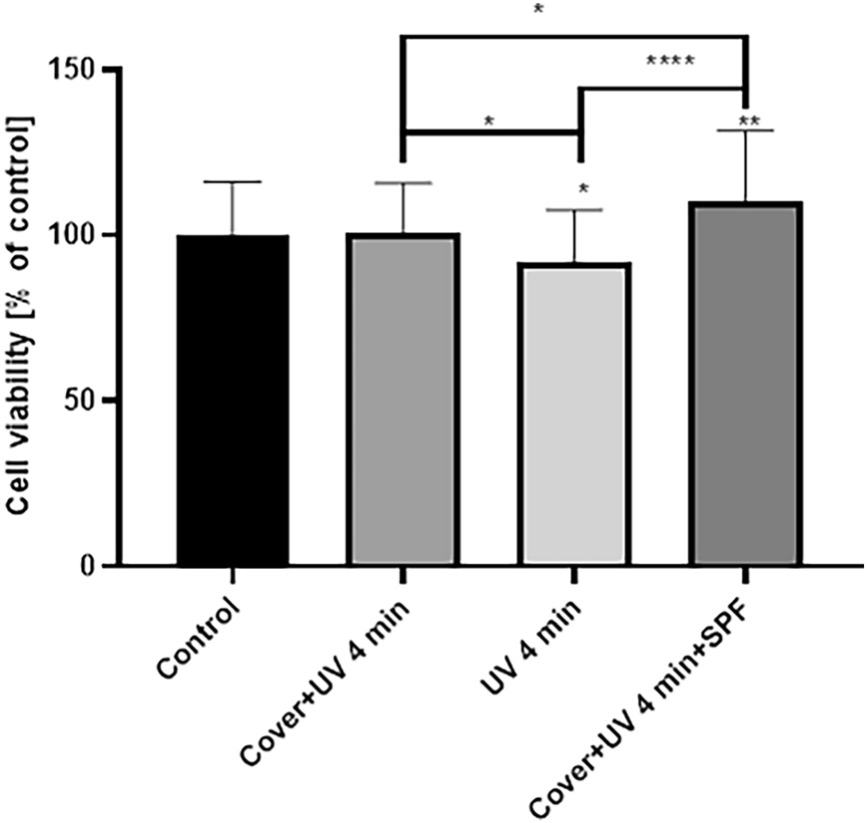
Cell viability of HaCat cell treated with 4 min of UV light. Values are mean ± SE of three independent experiments. Data were analyzed with GraphPad Prism Software version 8.0.1 using bidirectional ANOVA with Tukey’s multiple comparison tests; *p < 0.1, **p < 0.01, ****p < 0.0001 versus control. The results for the ANOVA test were: *p* < 0.0001, F = 11.43, total degree of freedom = 239.

Compared to the control, cell viability after irradiation with a UV lamp for 4 min decreased insignificantly by 8% (*p* < 0.1). In contrast, cells treated with light for 4 min with the lid covered with sunscreen showed 10% increased viability compared to the control (*p* < 0.01). Furthermore, cells irradiated for 4 min compared to cells irradiated with SPF protection show significantly decreased viability, with a difference of 19% (*p* < 0.0001). No statistical significance was demonstrated when comparing cells irradiated with UV light for 4 min with cells exposed to light and covered with a lid. (Fig. 2).

### In the case of treating HaCat cells for 20 min with UV light, the results were as followed

**Figure 3:**
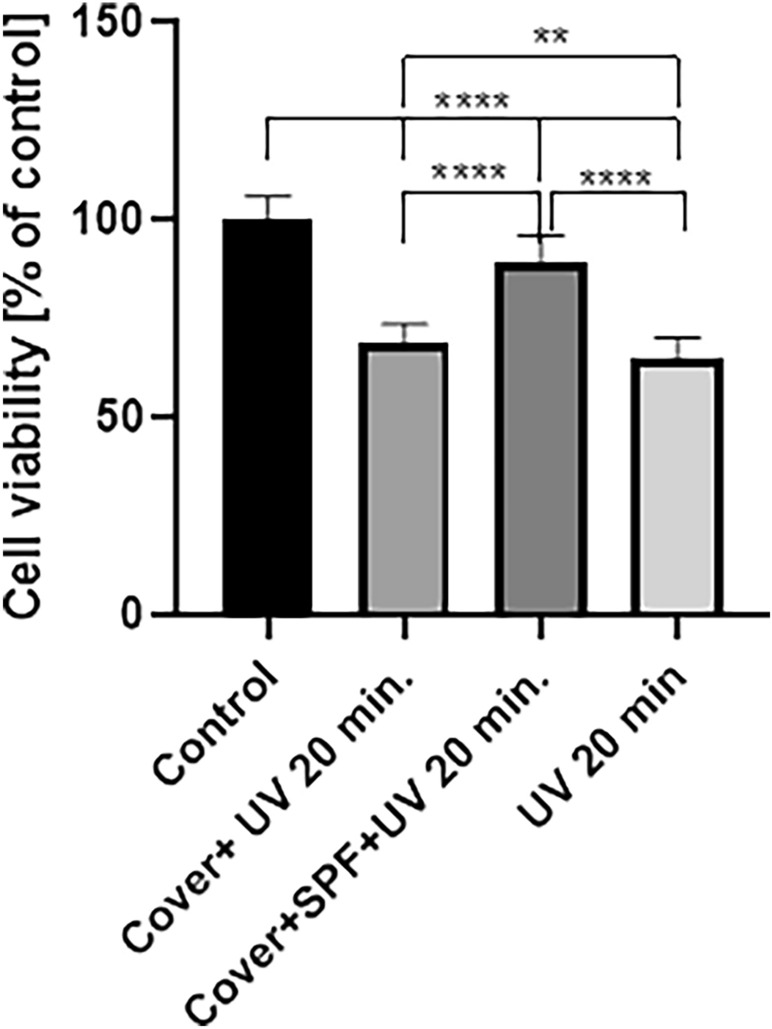
Cell viability of HaCat cells treated with 20 min of UV light. Values are mean ± SE of three independent experiments Cell viability assy. Values are mean ± SE of three independent experiments. Data were analyzed with GraphPad Prism Software version 8.0.1 using bidirectional ANOVA with Tukey's multiple comparisons tests; **p < 0.01, ****p < 0.0001 versus control. The results for the ANOVA test were: *p* < 0.0001, F = 511.1, total degree of freedom = 239.

Cell viability after irradiation with a UV lamp for 20 min without and with a lid decreased significantly by 35% and 31% respectively, compared to control (*p* < 0.0001). There was no significant difference between the viability of the cells irradiated with the lid and those without. Cells treated with UV light for 20 min with the lid covered with sunscreen cream showed a 10.5% reduction in viability compared to the control (*p* < 0.0001). Moreover, comparing the viability of cells protected with sunscreen cream to cells irradiated only with and without a lid, in both cases an enhanced viability of more than 20% was observed (*p* < 0.0001). (Fig. 3.)

## Discussion

The study was investigated in response to a recently published article by Maria Zhivagui et al., according to which there is strong evidence suggesting that the radiation emitted by UV nail dryers may cause cancers of the hand and that UV-nail polish dryers, similar to tanning beds, may increase the risk of early-onset skin cancer^7^. In our study, the human keratinocyte cell line HaCaT was exposed to UV for 4 min, which corresponded to the real conditions of typical exposure time per manicure being around 5 min per hand by UV nail polish dryers^10–12^. The cell viability of HaCaT cells after 4 min of radiation was 92% compared to the control, which is an insignificant reduction. After 20 min of irradiation, which does not mimic real conditions but is the minimum duration used in the study by Maria Zhivagui et al. cell viability was significantly lower than in the control group (*p* < 0.0001). This shows the importance of which UV irradiation duration is used. The results obtained could vary depending on the power of the UV nail drying device, however, comparable 48 and 56-W devices were used in both studies^7^.

In the literature, the safety of the use of UV lamps remains controversial. Nonetheless, most research assesses risk as low. Both studies, that of Diffey and that of Markova et al. evaluated the risk of squamous cell carcinoma (SCC) development from exposure to UVA nail lamps as very low^1,3^. Markova et al. compared the irradiance of UV nail lamps with exposure to narrowband UVB (NBUVB), which is used for phototherapy, and found that each of the three UV nail lamps studied produced a small fraction of the exposure of a single NBUVB run and therefore did not pose a clinically significant increased risk for developing skin cancer. Diffey ran a mathematical model combining age and UV exposure to estimate the risk of developing skin cancer from exposure to UVA nail lamps. According to the data obtained, tens or hundreds of thousands of women would have to use UVA nail lamps daily to develop SCC directly on the back of their hands. Additionally, John C Dowdy et al. performing a photobiological safety evaluation of six lamps, concluded that there is a maximum moderate risk during 29.8–276.25 min of permissible daily exposure^15^. Undoubtedly it is worth underlining the study conducted by Stern et al.^13^ who aimed to determine to measure the amount of UVA and UVB penetrating the nail plate using a radiometer and compared with a control. The results revealed that all studied fingernails completely blocked the UVB light, reading 0 mW/cm(2) on the radiometer, while the mean penetration of UVA light through the fingernails was 1.65%, ranging from 0.56% for the right fifth digit to 2.43% for the left second digit. The study proved that fingernails have the ability to protect the subungual skin against skin lesions induced by UV.

Despite ambiguous data on the safety of UV lamps, both the FDA and the authors of the studies recommend sunscreen with an SPF of 15 or higher application 30 min before lamp use and the provision of UV protective gloves^11,16–18^. The fact that sunscreen can be of great importance in protecting the skin when using a UV lamp is also confirmed by the results of our study. The viability of HaCat cells protected with sunscreen was significantly higher than without, with a difference of 20% and 30% for 4 and 20 min of radiation respectively (*p* < 0.0001).

In the literature, there is a lack of case studies describing the development of skin cancers after UV nail-drying device radiation. Squamous cell cancer (SCC) was linked to three cases after exposure to UV nail lamps. The time between exposure to UV light and the diagnosis of SCC varied between 11 and 15 years. SCC was confined to the dorsum of two women's hands and one woman's fingers. Two patients had several actinic keratoses, mostly on the same SCC sites. The other photo-exposed regions showed no symptoms of sun damage^2,19^.

The summary of major published studies suggests a low risk of skin cancer from nail lamps. However, the mixed results of the literature data were easily translated into the popular media, leading to confusion among customers and the industry towards UV nail polish dryers. According to a survey of 424 people conducted by Bollard et al.^10^ 72% of respondents believed that a UV nail lamp posed a cancer risk, and four out of five participants (82%) said they would not perform a gel manicure if they were aware of such associated risk. It shows that not only healthcare providers are confused, but patients too, which underlines the high need for further reliable research on this subject.

### Limitations and future directions

An important factor distorting the interpretation of research results in this area may be the lack of legal regulations and specific requirements regarding the selection of the power of UV/LED lamps and the wavelength generated by these lamps for specific products used in nail styling (gels, acrylgels ang gel polishes). The parameters of lamps offered by different manufacturers may differ, but sellers often do not provide information on how long products of other brands should be hardened in specific lamps. In the future, it is definitely worth performing an in-depth analysis of the lamp power in the first phase of its use and after 6 and 12 months of its use and examining the length of exposure time depending on the degree of pigmentation of the product and the formulation of specific materials from various manufacturers.

## Conclusions

This study shows that 4-min irradiation with UV nail polish dryers does not significantly reduce the viability of human HaCaT keratinocytes. Moreover, it shows that sunscreen with SPF 50 significantly increases cell viability compared to irradiated cells without cream. Many studies have shown that nail lamps carry a low risk of developing skin cancer, and the recently published study by Maria Zhivagui et al. does not replicate the exact conditions of the procedure. The study shows that even the minimal time of 20 min used in the paper cited above significantly alters the research results compared to 4 min of UV irradiation, which corresponds to real-world conditions. In the future, more extensive epidemiological studies are needed to accurately assess skin cancer risk in users of UV nail polish dryers. However, for this to happen, it is critical that the methods become more accurate and that the study conditions match real-world conditions. Our study indicates the efficacy of the use of sunscreen in protecting against UV light, which confirms the FDA recommendation to use sunscreen with an SPF of 15 or higher application 30 min before UV lamp use^18^.

## Data Availability

All data generated or analyzed during this study are included in this published article.
